# Lamotrigine-associated toxic epidermal necrolysis

**DOI:** 10.12669/pjms.39.6.7513

**Published:** 2023

**Authors:** Omair Farooq, Akifa Abbas, Muhammad Ahmad, Abu Bakr Manzoor

**Affiliations:** 1 Omair Farooq Assistant Professor, Medicine Department, Farooq Hospital, Lahore, Pakistan; 2 Akifa Abbas Demonstrator, Pharmacology Akhtar Saeed Medical & Dental College, Lahore, Pakistan; 3 Muhammad Ahmad Demonstrator, Pharmacology Akhtar Saeed Medical & Dental College, Lahore, Pakistan; 4 Abu Bakr Manzoor Demonstrator, Pharmacology Akhtar Saeed Medical & Dental College, Lahore, Pakistan

**Keywords:** Toxic epidermal necrolysis, Bipolar disorder, Lamotrigine

## Abstract

Toxic Epidermal Necrolysis (TEN) is an uncommon, idiosyncratic, potentially fatal dermatologic emergency affecting skin and mucous membranes. It is characterized clinically by blistering and widespread exfoliation, and pathologically by apoptosis of keratinocytes and epidermal necrosis. Drugs are one of the most common causative agents. The management ranges from nutritional support, care of denuded skin and mucosa to intravenous immunoglobulins (IVIG). The patient being reported developed TEN, secondary to lamotrigine; an anti-epileptic drug also used for bipolar disorder. On admission, lamotrigine was discontinued, he was managed symptomatically and given IVIG for three days. His condition started to improve after the first dose and he got discharged on 6^th^ day of admission.

## INTRODUCTION

Toxic epidermal necrolysis (TEN) is one of the rare acute blistering diseases characterized by mild to life threatening immune mediated dermatological exfoliative dermatitis affecting the skin conjunctival, nasopharyngeal, genital, and vaginal mucosa.1 The incidence of TEN is around 2.6 to 6.1 cases per million people per year with a mortality rate of around 5%. TEN is an aggressive form of Steven-Johnson syndrome involving more than 30% of the total body surface area (BSA). 2

A wide spectrum of mucocutaneous characteristics include erythematous, maculopapular, vesicular rash which subsequently progress into diffuse exfoliation of skin. It can also damage internal organs such as lungs, liver, kidney and cause hematologic or pulmonary impairment.3 Initiating factor for keratinocyte damage and apoptotic system are reactive oxygen species (ROS). TEN can happen as a result of drugs (Lyell syndrome), infections like staphylococcal toxins or can be idiopathic. Medications include Antibiotics, Anti-epileptics, NSAIDs, Ampicillin, Allopurinol, and Antiretroviral drugs. Anticonvulsants associated with TEN include the following Phenobarbital, Phenytoin, Carbamazepine, Valproic acid and Lamotrigine (LTG).4

The culprit drug may interfere with the detoxification pathways resulting in the accumulation of (ROS), triggering apoptosis. Lamotrigine is a widely used second line anti- epileptic drug also used in the treatment of neuropsychiatric disorder e.g.: bipolar disorder and neuropathic pain. The patient usually develops fever and other flu-like symptoms due to abnormal immune response one to three weeks after being exposed to medication followed by painful erythematous to purpuric skin lesions that tend to coalesce. Early recognition and treatment of this condition can alter the progression of the disease and save the life of the patient. Histological features include extensive full thickness epidermal necrosis with subsequent epidermal detachment.5 Once diagnosis gets established offending drug is promptly withdrawn. Supportive therapy and adjunctive therapies are then considered for treatment.^6^

## CASE REPORT

**Fig.1:**
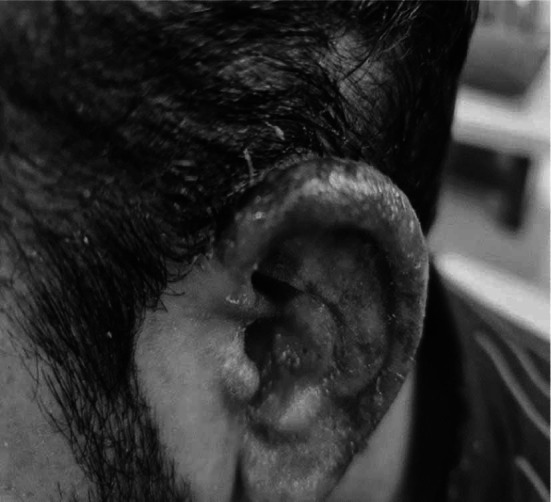
Erythema and erosion of ear skin at the time of initial presentation.

**Fig.2:**
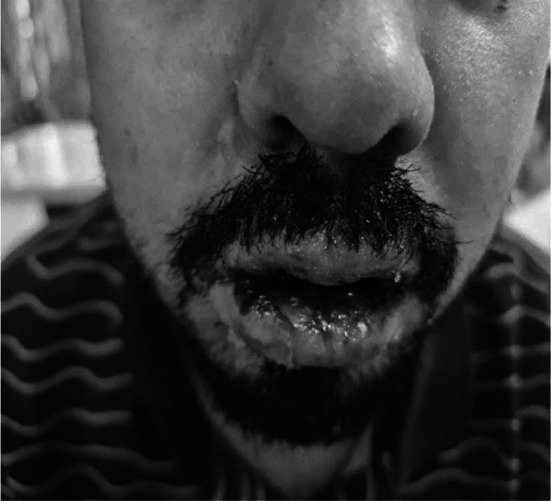
Crusted erosions and ulceration on lips and Oral cavity at the time of initial presentation.

A 27-year-old male patient presented in the emergency department of Farooq Hospital (west wood branch) with the complaint of fever, sore throat, and generalized rash for one week. He was a known case of bipolar disorder for the last five years and was on multiple medications for it, including Quetiapine, Valproic acid, Procyclidine and Benzodiazepines. His psychiatric consultant started him on Lamotrigine 50mg daily around seven weeks back. Almost one week back he developed rash on the arms, legs, feet, ankles, palms, front and back of the abdomen and chest along with the involvement of eyes and ears (Fig.1). On examination rash was erythematous morbilliform involving 60% of body surface area, with a positive Nikolsky’s sign. Conjunctiva was congested, oral mucosa showed mucositis and crusted erosions over lips. (Fig.2). Since Patient was taking Lamotrigine, a provisional diagnosis of Toxic Epidermal Necrolysis secondary to drug Lamotrigine was made. Drug was immediately withheld and the patient was admitted in the hospital. He was kept nil per oral as he was not able to swallow anything. Since he presented with fever, all of his cultures (blood, sputum, urine) were sent.

He was started on broad spectrum antibiotic Imipenem 500mg thrice a day. IV Hydrocortisone was started which was withheld after one day. Patient could not swallow and was immediately started on intravenous fluids. Immunoglobulin 1g/kg/day (patient weighed 75kg and dose of 75g/day) was started for a period of three days. The course was completed, and patient started showing signs of improvement after first dose. The rash started to regress, oral lesions started to become better, the erythema and congestion in the eyes improved, along with that the itchiness and redness of the rash became less and rash started to settle. Emollients such as Lubrex gel and Liquid paraffin were added to be applied topically.

Patient was asked to do gargles with normal saline. Imipenem was withheld once pan cultures were negative, Sucralfate and IV Omeprazole twice daily were added to control the gastrointestinal symptoms along with Dimenhydrinate and Ondansetron for the control of nausea and vomiting, IV Paracetamol SOS was added to control fever, the patient was administered intravenous Ringer’s lactate and Dextrose saline for IV hydration. Patient’s oral intake improved after the course of IV Immunoglobulin and was started on liquids and semisolids and the patient was discharged on 6^th^ day after admission, with follow up advice after one week.

**Fig.3:**
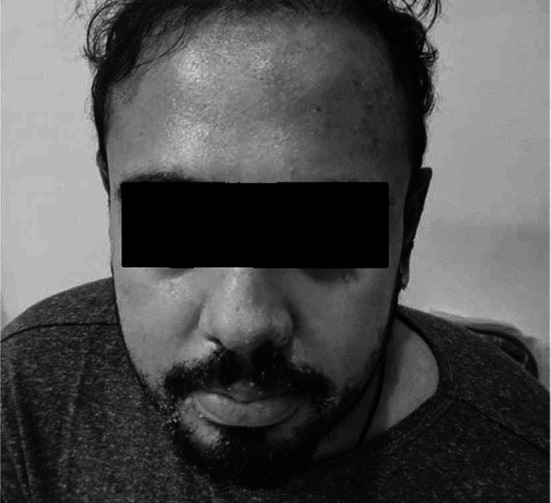
Follow up visit, one week after discharge.

**Fig.4:**
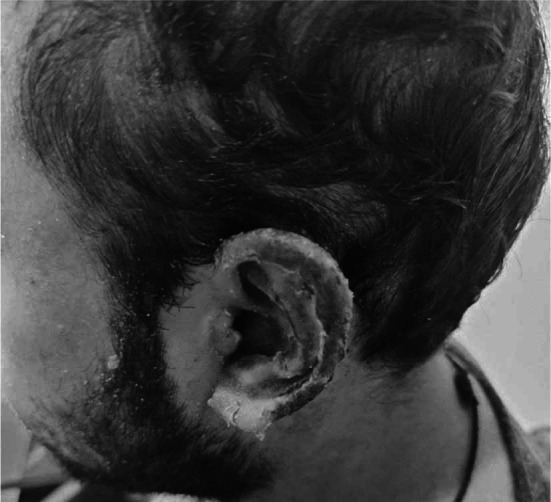
Ear skin has healed with crusting of skin and no erythema.

Although the patient was also on Valproate since last five years and that too is a cause of Toxic Epidermal Necrolysis, it seemed less likely being a cause as it was ongoing since last so many years but still it was withheld until complete recovery of the patient and agitation was controlled with Lorazepam which was continued for one week and then gradually tapered off. Lorazepam was then advised SOS after two weeks. Quetiapine was also continued at double dose. One week after discharge on follow up the patient generally appeared well, the mucositis and crusted erosions around the oral cavity improved significantly (Fig.3). The erythematous maculopapular rash on the body and eroded, ulcerated skin of ear healed with crusting (Fig.4).

## DISCUSSION

Skin manifestations as a result of drug reaction are common. Toxic epidermal necrolysis manifests as an acute life-threatening condition where erythematous lesions can be seen alongside epidermal necrosis. Full-thickness detachment of epidermis, mucosal membrane erosion exposing red dermis, and severe constitutional symptoms occur as a part of this drug- related skin disease. The toxic epidermal necrolysis is distributed along the face and upper body. It may spread across the entire body in a matter of hours.

There are several known causes of TEN which can broadly be classified into iatrogenic, infectious, and idiopathic. Adverse drug reactions are the commonest cause of TEN with more than 80% cases of TEN being drug induced. Toxic Epidermal Necrolysis is almost identical to Stevens Johnson Syndrome (SJS) but with respect to the current guidelines more than 30% of skin area involvement is considered as TEN whereas less than 10% involvement is classified as erythema multiforme and between 10 and 30 percent involvement is classified as Steven-Johnson Syndrome.^7^ Complications in TEN include hematuria, leukopenia, sepsis, and acute respiratory distress.

Lamotrigine is an antiepileptic drug that belongs to the Phenyl triazine class. After getting approved by the FDA, it was used as adjuvant therapy or monotherapy for adults or children with partial or generalized tonic-clonic seizures. Later, it was also approved as maintenance therapy for bipolar disorder. Lamotrigine is known to produce adverse effects consisting of symptoms like fever, skin rash, and multiple internal organ dysfunction. In a recent review of 3015 patients on lamotrigine, serious rashes in hospitalization occurred in eight patients. According to several previous studies, the risk factors that have been recognized to cause lamotrigine associated TEN include: administration of dose greater than the recommended initial dose, co-administration of lamotrigine with valproate, use of lamotrigine in patients younger than 13 years, and previous positive history of hypersensitivity reaction from another antiepileptic drug.^8^

According to the guidelines, management of patients diagnosed with TEN remains similar to thermal burns. The patient should be managed in an intensive care unit or burn center. All the baseline laboratory tests should be carried out, which also includes the liver enzyme assay, urinalysis. General supportive management also includes discontinuation of the offending drug, fluid and nutritional care, care of skin lesions with dressing in an attempt to prevent the secondary bacterial infections, and pain control.^9^ Systemic corticosteroids should also be administered to speed up the healing process, and there is no clinical contraindication for use against it.^10^ Plasmapheresis, cyclosporine, cyclophosphamide, and monoclonal antibodies have also been administered.^11^ In crux, the outcome of the patient significantly depends on the quality of medical care provided.

Our patient developed TEN seven weeks after he began lamotrigine therapy. Previous case reports implicated the combination of valproate and lamotrigine causing TEN, however, our patient had been taking valproate for almost five years. We believe lamotrigine was possibly the cause of TEN in our patient because the patient was started with a high dose of lamotrigine (50mg/day rather than 25mg every other day as recommended for use with Valproate).^12^

## CONCLUSION

It is of great importance that clinicians be aware of the potentially fatal adverse effects of taking lamotrigine and as reflected in previous case reports, combining lamotrigine with valproate leading to fatal reactions. Abiding by the escalation schedules described in the package labeling also reduces the frequency of lamotrigine-induced reactions. It is also important to note that, although it is hard to predict the possibly life-threatening reaction, the agent should immediately be discontinued at the first sign of a progressive rash.

### Authors` Contribution:

**AA, MA, ABM** data collection and manuscript writing.

**AA** edited, reviewed and did final approval of manuscript.

**OF** conceived and drafted the manuscript.

**MA** is responsible for the accuracy of the study.
